# Application of Contrast-Enhanced Ultrasound (CEUS) in Lymphomatous Lymph Nodes: A Comparison between PET/CT and Contrast-Enhanced CT

**DOI:** 10.1155/2019/5709698

**Published:** 2019-01-23

**Authors:** Xuelei Ma, Wenwu Ling, Fan Xia, Yifan Zhang, Chenjing Zhu, Jialing He

**Affiliations:** ^1^Department of Biotherapy, Cancer Center, State Key Laboratory of Biotherapy, West China Hospital, Sichuan University, Chengdu 610041, China; ^2^Department of Ultrasound, West China Hospital, Sichuan University, Chengdu 610041, China; ^3^West China School of Medicine, West China Hospital, Sichuan University, Chengdu 610041, China

## Abstract

**Purpose:**

We described imaging characteristics of different types of lymphomas using contrast-enhanced ultrasound (CEUS) and summarized some simple criteria to distinguish between normal lymph nodes and lymphomatous lymph nodes for clinical diagnosis.

**Materials and methods:**

Sixty-one lymphoma patients from 2014 to 2015 with 140 suspicious lymph nodes, who had been confirmed by histology and underwent chemotherapy, were enrolled in our study. The responses to chemotherapy were recorded by PET/CT, contrast-enhanced CT, or CEUS.

**Results:**

We summarized the CEUS enhancement patterns as two types when detecting lymphomatous lymph nodes, which could be the specific diagnostic criteria: (1) rapid well-distributed hyperenhancement, with 83.1% lesions exhibiting a fast-in hyperenhancement pattern in the arterial phase, and (2) rapid heterogeneous hyperenhancement, with 16.9% lesions exhibiting heterogeneous in the arterial phase. Particularly, we found that all the suspicious lesions of indolent lymphomas were rapid well-distributed hyperenhancement. CEUS successfully identified 117 lymphomatous lymph nodes, while PET/CT and contrast-enhanced CT detected 124 and 113 lymphomatous lymph nodes, respectively. CEUS had an accuracy of 83.57%, and the accuracy of PET/CT and contrast-enhanced CT was 88.57% and 80.71%, respectively (*p*=0.188). The false-negative rate was 16.43%, 11.43%, and 19.29%, respectively (*p*=0.188).

**Conclusion:**

CEUS could be a useful tool in detecting lymphomatous nodes. A rapid well-distributed hyperenhancement pattern in CEUS could be a useful diagnostic criterion in both aggressive lymphoma and indolent lymphoma. These results can help us distinguish between lymphomatous and benign lymph nodes and make better diagnostic and therapeutic decisions.

## 1. Introduction

Superficial lymphadenectasis is very common in lymphoma patients [1, 2]. Traditional imaging including conventional ultrasound (US), computed tomography (CT), and magnetic resonance imaging (MRI) mainly rely on the size and distribution of lymph nodes [3]. Unfortunately, the application of these methods is not ideal in clinical practice, as physicians cannot distinguish between benign and malignant nodes solely based on size parameters [4]. However, features in ultrasound could help identify lymphomatous lymph nodes based on the size, shape, and internal architectures [5]. For regional lymph nodes involved, all screening methods mentioned above display a fine sensitivity. Furthermore, the sensitivity of US was significantly higher than that of CT and MRI. In addition, the specificity of US was close to that of CT and MRI [6].

Contrast-enhanced ultrasonography (CEUS) with an intravenous contrast agent is a noninvasive imaging which could help establish differential diagnosis of suspicious lymph nodes [7, 8]. CEUS has proven its unique value in diagnosing lymphoma. After a bolus of 2.4 ml, contrast agent is administered, two characteristic phases are observed, arterial and venous, which could last approximately three minutes before clearance. As in the other contrast-enhanced examinations, hyperenhancement in the arterial phase should be expected.

Nowadays, many literatures have reported that CEUS could be a promising diagnostic modality in differentiating between benign and malignant lymph nodes [9]. However, only a few studies reported the diagnostic performance of lymphomatous lymph nodes [10]. Therefore, the current research status of CEUS imaging in the diagnosis of lymphoma is not yet satisfactory, and further improvement on characteristics of CEUS imaging is necessary. In our research, we used different imaging modalities to diagnose lymph nodes including CEUS, contrast-enhanced CT, and PET/CT. In our study, we analyzed CEUS imaging features of lymph nodes from 10 types of lymphomas. In addition, we analyzed the accuracy of CEUS, PET/CT, and contrast-enhanced CT, respectively. To the best of our knowledge, it is the largest research describing the imaging characteristics of different types of lymphomas secondary to Wendl CM et al. [11]. We summarized simple diagnostic criteria of lymphomatous lymph nodes. The results could help us distinguish lymphomatous lymph nodes and make better diagnostic and therapeutic decisions.

## 2. Materials and Methods

The retrospective, single-center study was approved by the Ethics Committee of West China Hospital, Sichuan University. Special informed consents were obtained prior to study participation including the understanding of limitations of CEUS in routine screening. Between January 2014 and December 2015, sixty-one lymphoma patients (43 males and 18 females; median age, 50.8 years; range, 17–77 years) with suspicious enlarged superficial lymph nodes were enrolled in our research. Patients were divided into different groups according to their pathologic classifications based on the World Health Organization (WHO) classification of neoplasms of the hematopoietic and lymphoid tissues, published in 2008. All the patients underwent pathologic confirmation. Prior to chemotherapy, 140 suspicious lymphomatous lymph nodes were evaluated by CEUS, PET/CT, and contrast-enhanced CT. The diagnostic criteria of these imaging are shown in Table 1. Six of the enrolled patients has a history of coronary heart disease. Seventeen patients have hypertension. Eleven patients have diabetes mellitus. The rest of the participants reported no comorbidities. Detailed information of the staging of patients and different subtypes of lymphomatous lymph nodes is demonstrated in Table 2.

## 3. US and CEUS Examinations

A commercially available ultrasound scanner (iU22; Philips Healthcare, Bothell, WA) with a L9-3 high-frequency linear array probe (3–9 MHz) was the primary tool for our study. The suspicious region was examined using gray-scale Doppler US for suspicious nodes, which were then selected by CEUS. The contrast agent used in this study was SonoVue (Bracco, Milan, Italy). A bolus of 2.4 ml of the contrast medium SonoVue was suspended in 5 ml physiological saline. We focused on one lymph node per injection. The maximum of contrast by SonoVue is 3 minutes. Multiple injections were obtained to evaluate multiple lymph nodes. Immediately thereafter administering the contrast medium, real-time gray-scale ultrasound examination of the lymph nodes was conducted, and dynamic images and video were recorded. Finally, the ultrasound images were independently evaluated by two experienced sonographers under the same examination protocol. If the evaluation from the two sonographers contradict, images would be reviewed by a third sonographer until a consistent evaluation is made.

## 4. Image Acquisition

### 4.1. PET/CT Examinations

The whole-body ^18^F-FDG PET/CT examination was performed by the Gemini GXL PET/CT scanner equipped with a 16-slice CT (Philips Medical System, Cleveland, Ohio, USA). All patients fasted for at least 6 h before intravenous injection of 190–375 MBq of ^18^F-FDG (5.18 MBq/kg). Blood glucose was closely monitored and controlled to lower than 8.0 mmole/L at the time of examination. A low-dose CT (5 mm slice thickness; tube voltage, 120 kV; tube current, 40 mAs) was performed for attenuation correction and immediately followed by the PET emission scan without changing the position of patients. PET and CT images were acquired from the head to extremities.

### 4.2. Contrast-Enhanced CT Examinations

All of the contrast-enhanced CT images were acquired using Philips Brilliance 16-slice detector-row machines (Philips Healthcare, Cleveland, Ohio). CT scan data were acquired using the following parameters: 120 kVp; 200 mA; rotation time 0.5 seconds; pitch 0.891 to 1.235; collimation 64 × 0.625 mm. Intravenous nonionic contrast material (1.5–2.0 ml/kg, Iohexol: Beijing Beilu Pharmaceutical, Beijing, China) was administered via the antecubital vein, using a power injector (Stellant D, Medrad, Indianola, PA) with a rate of 2-3 mL/s. All examinations were performed before and after contrast agent intravenous administration.

## 5. Results

### 5.1. CEUS Findings in Lymphomatous Nodes

**Figure 1:**
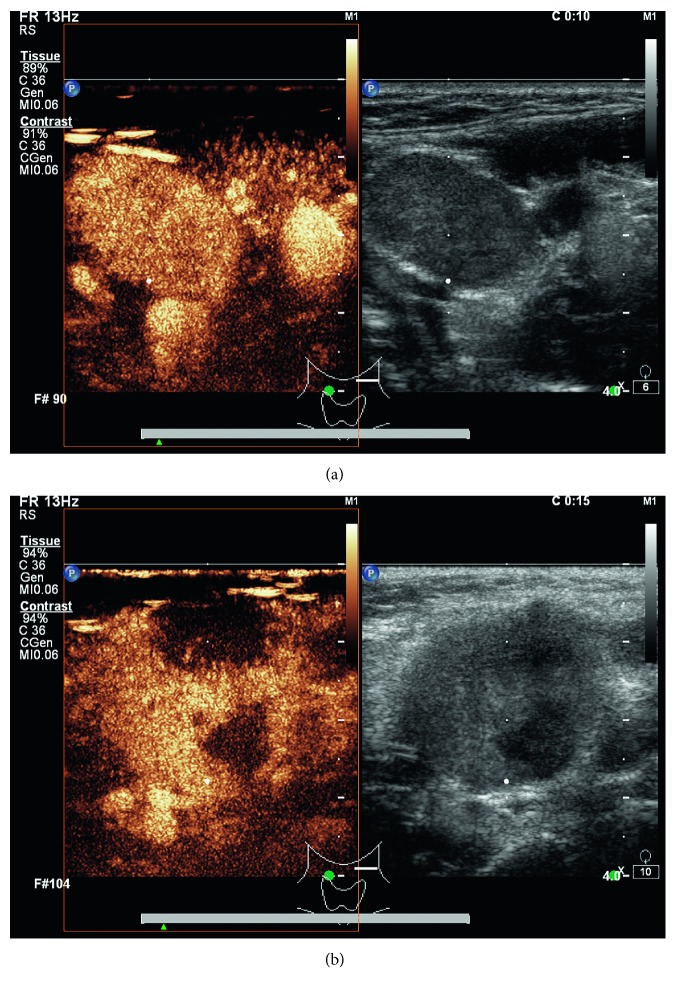
Sonogram obtained lymph nodes with a L9-3 high-frequency linear array probe (30 MHz) from different therapeutic states of lymphoma: (a) a lymphomatous lymph node with rapid heterogeneous hyperenhancement pattern; (b) a lymphomatous lymph node with rapid well-distributed hyperenhancement pattern.

In the lymphomatous nodes, one hundred and forty suspicious nodes were examined prior to chemotherapy including 79 nodes from diffuse large B-cell lymphoma (DLBCL) patients, two nodes from T-cell lymphoblastic lymphoma (T-LBL) patients, fifteen nodes from Hodgkin's lymphoma (HL) patients, sixteen nodes from peripheral T-cell lymphoma (PTCL) patients, one nodule from a anaplastic large-cell lymphoma (ALCL) patient, four nodes from mantle cell lymphoma (MCL) patients, eight nodes from Burkitt lymphoma (BL) patients, four nodes from inert small B-cell lymphoma (SBCL) patients, three nodes from diffuse large B-cell lymphoma (DLBCL) with follicular lymphoma conversion patients, and eight nodes from NK/T-cell lymphoma patients (Table 2). We compared all the images of lymphomatous nodes presenting fast-in (3–5 s) arterial phase with images of normal nodes or carcinoma nodes. We divided the enhancement pattern into 2 types based on the extent of enhancement, the uniformity of echo intensity, and the time of arterial enhancement: (1) “rapid well-distributed hyperenhancement,” with 83.1% lesions exhibiting the fast-in hyperenhancement pattern in the arterial phase, and (2) “rapid heterogeneous hyperenhancement,” with 16.9% lesions exhibiting heterogeneous in the arterial phase (Table 3; Figures 1(a) and 1(b)). However, examiners could not identify directions of blood flow in all the lymphomatous lymph nodes because they were either hybrid or centripetal.

In our study, we examined 140 superficial lymphomatous lymph nodes which had been evaluated by PET/CT and contrast-enhanced CT, in addition to CEUS. Among all the lymph nodes, CEUS detected 117 lymphomatous lymph nodes, while PET/CT and contrast-enhanced CT detected 124 and 113 lymphomatous lymph nodes, respectively. CEUS had an accuracy of 83.57%, while the accuracy of PET/CT and contrast-enhanced CT was 88.57% and 80.71%, respectively (*p*=0.188). The false-negative rate was 16.43%, 11.43, and 19.29%, respectively (*p*=0.188) (Table 4).

### 5.2. CEUS Finding in Indolent Lymphoma

Current research suggests that the accuracy of PET/CT in differentiating benign from malignant tumors is not ideal in indolent lymphoma. In our research, we enrolled 2 indolent lymphoma patients (Table 3). These patients were suspected due to abnormal contrast-enhanced CT findings and were later confirmed by pathological results. The diagnosis of these patients was inert small B-cell lymphoma. We found that all these suspicious lesions from the 2 patients were rapid well-distributed hyperenhancement on CEUS. Initial results suggested that there was no significant advantage in identifying aggressive lymphoma. Further researches could be done on the CEUS imaging characteristics of indolent lymphoma.

## 6. Discussion

Enlarged lymph nodes are the most common clinical symptom and chief compliant in a variety of lymphomas [12]. In our research, we summarized CEUS imaging patterns in lymphomatous lymph nodes prior to chemotherapy. Previous literature has reported that CEUS could evaluate tumor vascularity [13, 14]. Studies have shown that lymphomatous lymph nodes infiltration shares similar features with nonmalignant nodes. However, the details were never discussed. The European guidelines discussed the limitations of CEUS in lymphomas and did not recommend CEUS for routine use in discriminating between benign and malignant. The guidelines mentioned the limitations of CEUS including a demand for relatively bigger size of lymph nodes, while the fact is that about one-third of malignant lymph nodes is less than 5 mm. Hence, CEUS is only recommended in special settings [15]. However, in our clinical practice, we discovered that CEUS has its own advantage in lymphomas. In our study, we found that the arterial phase of enhancement in lymphomatous lymph nodes was classified as fast-in and nonfast-in. In addition, previous reports showed that time to peak of benign and carcinomatous lymph nodes was longer than surrounding tissues [11, 16]. These characteristics are helpful in making differential diagnosis of lymphomatous lymph nodes. In our research, we found the contrast agents produced a centripetal/hybrid or homogeneous/heterogeneous enhancement pattern in the arterial phase in lymphoma patients before treatment. And the homogeneous enhancement was found in the vast majority of cases. One case report of T-cell lymphoma presented a similar CEUS imaging [17]. Based on the above results, we can briefly summarize that the CEUS imaging of lymphomatous lymph nodes was “rapid well-distributed hyperenhancement” or “rapid heterogeneous hyperenhancement.” It is our opinion that CEUS is a promising tool for identifying lymphomatous lymph nodes.

The comparison between the three imaging modalities indicated there is no statistical significance between the accuracy of CEUS, PET/CT, and CECT. Several researches have discussed the advantages of CEUS over the other imaging modalities [18, 19]. One research showed that the sensitivity of detecting the metastasis of internal mammary lymph nodes in breast cancer was 96.7%, while 92.9% in CEUS and PET/CT, respectively [20]. CEUS could present lymph nodes images as well as PET/CT in regional lymph nodes of lymphoma patients [21]. In addition, CEUS has the advantage of being inexpensive and nonradioactive. Hence, it could be valuable in both diagnosing and monitoring the response to chemotherapy.

Our research points to a future research direction, which is the potential clinical application of CEUS on indolent lymphoma. PET/CT with FDG is not commonly used in indolent lymphoma as it is in Hodgkin lymphoma (HL) and diffuse large B-cell lymphoma (DLBCL) because FDG uptake is lower in indolent than in aggressive lymphoma. Recent studies have reported that indolent lymphoma patients could benefit from traditional functional imaging due to low sensitivity and specificity [22]. In our study, CEUS imaging of indolent lymphoma was only influenced by the blood flow, and two indolent patients with four highly suspected lesions were enrolled in our research. All these patients have been confirmed by pathological diagnosis, and these suspicious lesions demonstrated a rapid well-distributed hyperenhancement pattern on CEUS. It indicates that CEUS could offer a novel clinical perspective in identifying benign and malignant nodes in indolent lymphoma. The treatment and evaluation of indolent lymphoma still remains controversial. A major reason is that functional imaging could not perform well. We hypothesized that the region functional imaging of CEUS could offer some perspective on treatment and evaluation of indolent lymphoma patients.

Although this is the first study mainly focused on lymphomatous lymph nodes, our study has some limitations. Firstly, no normal patients enrolled in the study. As a retrospective study, we focused on the specific patterns of lymphomatous lymph nodes and tried to conclude the CEUS manifestation pattern. Secondly, CEUS is not an individual baseline detecting method and often used as a regional screening method which provides important functional information in the suspected region, while advanced lymphoma could distribute all over the body. Thirdly, compared with PET/CT and contrast-enhanced CT, CEUS imaging can be disturbed by gas or bone interferences, which make it only sensitive in detecting superficial, but not in deep layers. If the lymphomatous nodes were in the mediastinal lymph node area, close to the intestinal wall area or under bone structures, CEUS would be blind to the lesions. Fourthly, while we try to screen as much pathological types as we can, some relatively rare types of lymphoma were absent. Fifthly, due to the low occurrence rate of indolent lymphoma, we could not establish the criteria for diagnosis.

## 7. Conclusion

In conclusion, our study summarized the imaging characteristics of CEUS in the lymphomatous nodes. Rapid well-distributed hyperenhancement could be a useful criterion both in diagnosing aggressive lymphoma and indolent lymphoma. These results suggest that CEUS has a potential diagnostic value to detect suspicious lymphoma noninvasively, and it may also help in future clinical practice.

## Figures and Tables

**Table 1:** Criteria of the imaging method including CEUS, contrast-enhanced CT, and PET/CT to diagnose the malignant lymphomatous nodes.

Imaging modalities	Criteria for diagnosis of lymphomatous lymph nodes
CEUS	Rapid well-distributed hyperenhancement lymph nodes Rapid heterogeneous hyperenhancement lymph nodes

PET/CT	Positive lesion shown as focal or diffuse FDG uptake above background in a location incompatible with normal anatomy or physiology without a specific SUV cutoff SUV_max_ > 14 informs the lymph nodes had transformed, whereas those with SUV_max_ < 14 do not

Contrast-enhanced CT	Maximum diameter is ≥10 mm, and minimum diameter is ≥6 mm Central area of neuroses Margins are blurred The target metastasis lymph nodes have special reinforcement

**Table 2:** Clinical characteristics of lymphomatous patients.

Classification	Number of patients (*N*)	Number of lymphomatous lymph nodes (*n*)
Gender		
Men	43	115
Women	18	25
Age		
>60	20	42
<60	41	98
Stage		
I	3	9
IE	2	5
II	14	33
IIE	11	24
III	12	26
IIIE	6	13
IIIS	7	17
IIISE	2	4
IV	4	9
Pathologic diagnosis		
*Precursor cell derived*		
T-cell lymphoblastic lymphoma	1	2
*Mature T-cell derived*		
Peripheral T-cell lymphoma	6	16
Extranodal NK/T-cell lymphoma	4	8
Anaplastic large-cell lymphoma	1	1
*Mature B-cell derived*		
Diffuse large B-cell lymphoma	34	79
Burkitt lymphoma	4	8
Mantle cell lymphoma	2	4
Inert small B-cell lymphoma	2	4
Diffuse large B-cell lymphoma (follicular lymphoma conversion)	1	3
* Hodgkin's lymphoma*	6	15
Total	61	140

**Table 3:** Enhancement pattern of aggressive and indolent lymphoma.

Classification of lymphoma	Fast-in	
	Rapid well-distributed hyperenhancement	Rapid hyperheterogeneous enhancement
Aggressive lymphomatous lymph nodes (136)	81.6% (111/136)	18.4% (25/136)
Indolent lymphomatous lymph nodes (4)	100% (4/4)	—

**Table 4:** The accuracy of CEUS, PET/CT, and CECT in detecting lymphomatous lymph nodes.

Classification	CEUS	PET/CT	CECT	*p*
T-cell lymphoblastic lymphoma (%)	2/2 (100)	2/2 (100)	2/2 (100)	—
Peripheral T-cell lymphoma (%)	13/16 (81.25)	15/16 (93.75)	14/16 (87.5)	0.859
Extranodal NK/T-cell lymphoma (%)	7/8 (87.5)	7/8 (87.5)	6/8 (75)	1
Anaplastic large-cell lymphoma (%)	1/1 (100)	1/1 (100)	1/1 (100)	—
Diffuse large B-cell lymphoma (%)	65/79 (82.28)	71/79 (89.87)	62/79 (78.48)	0.145
Burkitt lymphoma (%)	6/8 (75)	7/8 (87.5)	6/8 (75)	1
Mantle cell lymphoma (%)	3/4 (75)	4/4 (100)	4/4 (100)	1
Inert small B-cell lymphoma (%)	4/4 (100)	0/4 (0)	3/4 (75)	0.03
Diffuse large B-cell lymphoma (follicular lymphoma conversion) (%)	3/3 (100)	3/3 (100)	3/3 (100)	—
Hodgkin's lymphoma (%)	13/15 (86.67)	14/15 (93.33)	12/15 (80)	0.858
Total (accuracy, %)	117/140 (83.57)	124/140 (88.57)	113/140 (80.71)	0.188
False-negative rate (%)	23/140 (16.43)	16/140 (11.43)	27/140 (19.29)	0.188

## Data Availability

The data used to support the findings of this study are available from the corresponding author upon request.

## Abbreviations

CEUS: Contrast-enhanced ultrasound
US: Ultrasound
CT: Computed tomography
CECT: Contrast-enhanced computed tomography
MRI: Magnetic resonance imaging
PET/CT: Positron emission tomography/computed tomography
DLBCL: Diffuse large B-cell lymphoma
T-LBL: T-cell lymphoblastic lymphoma
HL: Hodgkin's lymphoma
PTCL: Peripheral T-cell lymphoma
ALCL: Anaplastic large-cell lymphoma
MCL: Mantle cell lymphoma
BL: Burkitt lymphoma
SBCL: Small B-cell lymphoma.
